# Evaluation of an aged care nurse practitioner service: quality of care within a residential aged care facility hospital avoidance service

**DOI:** 10.1186/s12913-017-1977-x

**Published:** 2017-01-13

**Authors:** Trudy Dwyer, Alison Craswell, Dolene Rossi, Darren Holzberger

**Affiliations:** 1 Central Queensland University, Building 18 Rockhampton, Bruce Highway, Rockhampton, Q 4702 Australia; 2 School of Nursing, Midwifery and Paramedicine, University of the Sunshine Coast, 90 Sippy Downs Drive, Sippy Downs, Q 4556 Australia; 3 Central Queensland Hospital and Health Service, Queensland Health, Rockhampton, Q 4770 Australia

**Keywords:** Nurse practitioner, Donabedian, Aged care facility, Geriatrics, Health service evaluation

## Abstract

**Background:**

Reducing avoidable hospitialisation of aged care facility (ACF) residents can improve the resident experience and their health outcomes. Consequently many variations of hospital avoidance (HA) programs continue to evolve. Nurse practitioners (NP) with expertise in aged care have the potential to make a unique contribution to hospital avoidance programs. However, little attention has been dedicated to service evaluation of this model and the quality of care provided. The purpose of this study was to evaluate the quality of an aged care NP model of care situated within a HA service in a regional area of Australia.

**Methods:**

Donabedian’s structure, process and outcome framework was applied to evaluate the quality of the NP model of care. The Australian Nurse Practitioner Study standardised interview schedules for evaluating NP models of care guided the semi-structured interviews of nine health professionals (including ACF nurses, medical doctors and allied health professionals), four ACF residents and their families and two NPs. Theory driven coding consistent with the Donabedian framework guided analysis of interview data and presentation of findings.

**Results:**

Structural dimensions identified included the ‘in-reach’ nature of the HA service, distance, limitations of professional regulation and the residential care model. These dimensions influenced the process of referring the resident to the NP, the NPs timely response and interactions with other professionals. The processes where the NPs take time connecting with residents, initiating collaborative care plans, up-skilling aged care staff and function as intra and interprofessional boundary spanners all contributed to quality outcomes. Quality outcomes in this study were about timely intervention, HA, timely return home, partnering with residents and family (knowing what they want) and resident and health professional satisfaction.

**Conclusions:**

This study provides valuable insights into the contribution of the NP model of care within an aged care, HA service and how staff manipulated the process dimensions to improve referral to the NPs. NP service in this study was dynamic, flexible and responsive to both patient and organisational demands.

## Background

Australia, similar to other developed countries, is facing unprecedented challenges to meet the growing healthcare needs of an aging population. It is predicted that by 2050, upwards of 3.5,000,000 Australians will be accessing aged care services annually. This growth will occur alongside a forecast health workforce shortage and a decreasing number of primary care physicians (PCP) visiting Aged Care Facilities (ACFs) [1]. In an effort to meet this increased demand, different healthcare service delivery models are emerging that challenge traditional professional health boundaries. Such models include the emergence of the nurse practitioner (NP), who is endorsed to provide specialised healthcare services such as prescribing medications, referral and ordering of specific diagnostic investigations [2]. The title of NP is protected by law, has rigorous accreditation processes and can only be used by those educated at Masters level and endorsed by the national registration body [3].

Australia has universally accessible funded healthcare. This government funded, public health insurance scheme (called Medicare) provides free or subsidised healthcare for services provided by health professionals [4]. Eligible health professionals, including NPs and medical doctors, are able to gain remuneration for specific services listed on the Medicare Benefits Schedule. This schedule provides a list of eligible health service rebates and a range of prescribed medications [5]. Australian NPs only have access to four items compared to the hundreds of items available for the medical practitioners, and reimbursement for the NP service remains low at 85% of the scheduled fee [5]. The context in which the NP practices determines their scope of the practice. Currently little is known about individual NP models of service delivery [6].

NP models providing services to the elderly are emerging in many formats. Models of care include NPs initiating independent private practice, being situated in the ACF, working from community-based organisations, or as an outreach service from the acute hospital setting as a member of a multidisciplinary team [7–11]. Specialist aged care services provided by the NP include dementia care [12, 13], management of delirium (in acute inpatient public hospital) [14] and nurse-led telephone support service to ACFs [15, 16]. Overall, these examples demonstrate NP-led models of care in aged care can provide holistic care, positively impact on residents’ physiological and psychological symptoms and quality of life by reducing hospital admissions [8, 10, 15, 16]. Whilst these variations to the NP models of care continue to develop, little attention has been devoted to the evaluation of the quality of care provided. The purpose of this study was to evaluate the quality of the NP model of care within a HA service in a regional ACF context [17].

### Donabedian model

The Donabedian model was chosen as the theoretical framework for the study as it considers the complex, multi-dimension aspects of a health service when evaluating the quality of the NP model of care [18]. Donabedian uses a systems perspective incorporating evaluations of structure, process and outcome (SPO) to evaluate health service delivery [19]. The SPO sub-dimensions represent the key components of the healthcare supply chain and have been used extensively to evaluate the quality of care and nursing care performance in many contexts such as the nursing handover process, wound care and NP assessment of chest pain in the emergency department [6, 17, 20–23]. The Structural element examines how care is organised and the characteristics that impact the ability of the nursing system to meet healthcare needs. Process elements are analysed considering the character of the practice environment and the nature of the activities undertaken in providing care [24]. The last dimension, Outcome, is described as the impact of the nursing care on the state of health and events that follow. Donabedian asserts that good structure increases the chances of good processes and good processes in-turn increase the likelihood of good outcomes [25]. The research question for this study was, ‘What health service structures influence the delivery of safe, quality care for residential aged care residents?’ The Donabedian framework was used to inform the research design, data collection and analysis to evaluate the relationships between the SPO dimensions of NP model of care, within an ‘in-reach’ hospital avoidance service.

## Methods

### Design

This paper reports on the qualitative component of a larger interpretative study that evaluated three distinct NP models of care (aged care, cardiac and respiratory) within aged care in a regional context in Australia.

### Setting of the study

The setting for this study was a community public health service in a regional setting of Central Queensland, Australia. The region has a total population of 112,300 with approximately 10% of this population over 70 years of age and living in an ACF [26]. The NPs in the study are positioned within a community-based ‘in-reach’, HA service called the Residential Acute Care Service (RACS). The RACS team who respond to calls from ACFs, is a responsive, mobile triage service that services ten public or privately operated facilities, with a total of 773 residential care beds. This team is not ‘attached’ to any one facility and consists of two fulltime equivalent NPs, who work office hours Monday through Friday, and two and a half fulltime equivalent registered nurses with advanced clinical experience (one on-call after hours). To be included in the study, users of the NP service were defined as residents or their families of the regional ACFs. The providers of the service were two NPs (one gerontology and one chronic disease endorsed), ACF nurse managers, registered nurses, allied health professionals and general practitioners who provided medical care to the residents.

### Ethical issues

Ethics approval was received from both the study site and the University Human Research Ethics Committees of the Hospital (ETHICS numbers HREC/15/QCQ/18, H15/03-040). Prior to data collection all potential participants were provided with a plain language information letter outlining the purpose, voluntary nature of the study and the process involved with collection of data. Only residents who were able to understand the information letter and provide fully informed consent were invited to participate in the study. Consenting participants signed a consent form.

### Data collection

The Australian Nurse Practitioner Study (AusPrac) standardised interview schedules [27, 28] guided the interview questions. Data were collected between July and October, 2015. Letters of invitation to participate in the study were distributed to a purposive sample of residents, family and health professions who interacted with the NP service. Interested persons contacted the research team, and a convenient time to participate in the interviews was negotiated. A total of 15 interviews were conducted; nine health professionals, four health care consumers and the two NPs.

### Data analysis

#### Interviews

All interviews were audio-recorded and transcribed verbatim. Researchers became immersed in the data by listening, reading and re-reading the transcripts. A hybrid, data-theory driven content analysis was adopted within the SPO framework [17]. At the first level, the research team listened to audio recordings, read and re-read the transcripts independently to determine recurring codes in the data. Next, a second round of theory driven coding occurred where the Donabedian quality framework (SPO) guided the analysis [25]. The team then reviewed, discussed codes, returning to the data as needed to substantiate and finalise the emergent themes (using the Donabedian dimensions). This approach ensured common understandings within the team. The entire iterative process of data collection, analysis and theory driven coding by the team ensured the quality and rigour of the study [29, 30].

## Results

The findings from our study are presented in accordance with the Donabedian meta-concepts of: structure, process and outcomes. The concepts are presented with excerpts of participant voice to illustrate examples of each concept [31].

### Structure

The structural dimensions of the NP service within the community based RACS is as an HA service model. When a resident is deteriorating, this model supports the NP to intervene early and, if the resident is admitted to hospital, to facilitate early discharge back the ACF.

*Our hospital avoidance strategy that we work under is responding to acute referrals and that’s the criteria that we’re meant to respond to for our referrals. Yes it is very reactive [NP].*

The NPs in this studywill attend to around 240 residents with around 1100 occasions of service annually. As the medical governance regarding the clinical treatment remains with the resident’s PCP, the NP liaises with the resident’s PCP to communicate their assessment findings, diagnoses, plan of care, treatments initiated and referrals as close as possible to the time of consultation. NPs also liaise with the senior medical officer in the emergency department (ED) about resident concerns and directions of treatment if it is an emergency situation, the PCP is unavailable or at the recommendation of the PCP. Unless otherwise negotiated, the NPs follow up each episode of care for up to 3 days.

Unique to this regional service is that the NPs respond to up to ten ACFs across several towns, which means they can spend a lot of their workday in the car travelling. For up to ninety percent of the referrals, the NPs reported traveling for 30 min or more to attend the resident.

*We’re always on the road to respond to our referrals so it's time limiting. We have a good turnaround. Our referrals we probably see within a 24 h period, generally within 12 h…we see them very quickly, so just the mere fact that we’re a mobile service, I guess, is limiting in some situations [NP]*.

The mobile ‘in reach’ service means the NPs have to travel with all necessary equipment to respond to a variety of situations. This can be heavy work *“taking the hospital to the person”.* When a resource is missing or required the NP have to travel back to the office, which is time consuming.

### Process

The process dimensions within the SPO framework that were manipulated to improve the quality of the service [32] were categorised/identified as; the referral process, the response process and the flow process.

### Referral process

The process of referral involved the ACF staff assessing the resident and requesting an internal review by either the RACS team or the clinical nurse at the facility. The decision was then made to refer the resident to their PCP, the NP, or transfer the resident directly to the Emergency Department (ED). This second step in the referral process is ad-hoc and context specific with little apparent adherence to the formal referral structure.

*We sort of swing now … oh I suppose if we can’t contact the GP we will offer him a fax and then we will send him a fax outlining our queries, concerns with the resident and then we will try to contact the Nurse Practitioner. So they put a note on that something is happening, if they cannot get there in an awful hurry, with 9 times of 10 they can’t. So we normally will ring up the Nurse Practitioner and they can give us some strategies over the phone [RN]*.

The chosen referral process was primarily driven by the desire of the ACF staff for a timely referral and response to their identified concerns. The typical referral process for contacting the PCP was to send a fax, email or call the practice directly, a process that is often either delayed or lacking. Competing priorities are evident as the practice nurses and/or receptionists ‘triage referral requests’ prior to bringing it to the doctor’s attention. Not being able to speak directly to the PCP or having a common process for triaging referral requests is a limitation of the current referral process. Additionally the PCPs, because of competing practice priorities, often respond after the practice needs are met, either after hours or early in the morning.

*So a GP (doctor), because of their workload might say ‘well I can’t get out there for 24 h, oh well that is just the way it is going to be’. And that is how it happens. So there is a delay in care [RN]*.

Acknowledging the delay, ACF staff then decide to either wait for the PCP to respond, transfer to the ED (occasionally at the request of the PCP) or to contact the NP. Trust, knowledge of the NP role and traditions influence this decision. Residents and ACF staff often have long standing relationships with their PCPs and it is loyalty to this relationship that influences the referral process.

*It can be for some people, from my experience just having a longstanding relationship, more of a friendship with the GP (doctor] [RN]*.

Some PCPs may choose to send the resident directly to the hospital, because of perceived professional boundaries;

*…some of my colleagues [medical doctors] … don’t like their treatments challenged or their diagnoses challenged [PCP]*.

Participants believe these relationships alone should not determine the referral process as referral to the PCP did not always result in the optimal outcome for the resident.

*You are better off having something done than nothing, you know, you institute a treatment and a management (plan) earlier than if you have had to wait. Like, if I had to wait until 6 o’clock at night to go and see someone who should have been seen six hours prior, it just sets their …. That whole thing back even more so that you end up sending someone to a hospital for admission at 6 o’clock at night rather than at midday. It just does not make sense [PCP]*.

PCPs who participated in this study were accepting and grateful for the contribution of the NP service. Knowledge of and prior exposure to the NP role influenced the referral process contributing to unnecessary resident transfer to ED.

*The NP has explained to the staff members that even if it’s, you know, if there’s a laceration or something that needs fixing and it happens in the middle of the night, sometimes it can wait until the next day and the NP see them rather than them being shipped to the ED in the middle of the night, but, yeah, sometimes I think they [ACF staff] just panic maybe or lack of education from the nursing home perspective [RN]*.

One participant proposed that, to reduce the number of unnecessary ACF facility resident transfers to the ED, all residents should have a mandatory formal review process by the NP prior to transfer to the ED.

*I would like just for there to be a mandatory step somewhere in there that the NP reviews the patient before transfer to hospital because a lot of the time it's just the GP (doctor) says “I can’t come to the facility, send them to hospital” and it might very well be something that the NP could have managed without the transfer. But sometimes, well a lot of times, that gets missed [Other health professional]*.

The ACF team will refer directly to the NP service when; 1) the PCP is on leave; 2) the resident requires palliative care; 3) the ACF staff are seeking a nursing response or; 4) the residents family specifically request the NP response. As the role and scope of practice of the NP is emerging, ACF staff are beginning to consult with the resident and/or their family prior to making the decision to refer to either the NP or the PCP.

*Um, well initially it can be patient choice (to call the NP). So a lot more consumers are knowing the role of the Nurse Practitioner, particularly within residential aged care. … So, um, we also give patients a choice. They understand though that we will collaborate with the GP (doctor). So it just depends what their needs are [RN]*.

and

*Some residents have requested regular care from the NP's instead of their GP (doctor/PCP) as they feel they are more attentive and have better communication skills [ACF manager]*.

Being known impacts on interprofessional trust and the referral process. However, building trust takes time, and this is more apparent in regional area where most health professionals know each other. The following extract show how acceptance of formal qualifications was not sufficient;

*…it is a matter of trust and particularly in regional areas. I think everybody knows everybody so there is an unwritten need to prove yourself before people hand over…and I don’t believe it matters what qualification you are, I think that it’s just the way that it is in the country [NP]*.

The structural dimension of NPs work hours limits the availability of the NP to respond or accept referrals both afterhours and on weekends. At these times, aged care staff resort to either referral to the PCP or transferring the resident to the ED for review.

### Response process

The emergent themes used to describe the response process are: responding with advanced scope of practice and responding within a nursing model of care.

#### Advanced scope of practice

The NPs, because of their extended clinical scope of practice, prescribing and referral authority [2] were able to respond and intervene in a timely and supportive manner, meeting the needs of the resident, family and ACF staff. In addition to undertaking advanced clinical skills such as changing difficult indwelling catheters and complex wound management, the NPs prescribed, referred residents to other health professionals, and delivered on-going staff development sessions. Participants reported that the ‘traditional biomedical’ interventions initiated by the NP were comparable to services provided by the PCP.

*Ummm … there’s been several occasions when people have had UTIs. They’ve [NPs] put them on medication. It’s resolved [RN]*.

Additionally, from the perspective of the PCP.

*Well I’ve even had the NP set up [blood] transfusions for me. I do transfusions on nursing care, nursing home patients. It’s a great service it works well [PCP]*.

NPs, when responding, spent time upskilling ACF staff and assisting with confirmation of the decisions they have made. The following statement is reflective of many ACF nurses.

*Um, I mean, end of life care is much better because they [NP] back us up with, like, the syringe drivers, blood transfusions we do here with their back up. Whereas normally all of that would … they [residents] would have to go to hospital for that [ACF RN]*.

NP support meant staff were more confident to keep a larger cohort of residents at home. This combination of working to full capacity, delivering optimal care and keeping residents at home contributed to ACF staff workplace satisfaction.

#### Nursing model of care

The NPs believed they functioned within a holistic, wellbeing framework focusing on improving quality of life, taking time to talk and listen to the resident and family to elicit their healthcare or end of life goals.

*So we take the time to assess everything, all of their concerns from the health perspective, and from a wellbeing perspective, to see if we can improve their lifestyle and improve their quality of life and even if that is just education or steering them in the other direction of healthcare services, that is what we’ll do [NP]*.

The following extracts reflect the two different approaches when attending the palliative needs of residents;

*… a lot of times in aged care the GPs (doctor/PCP) come and take this ‘set and forget’ approach. … they (doctor/PCP) kind of go ‘yeah their palliating okay, we will put them on end of life and yeah let me know when you need more drugs’ kind of thing [ACF RN]*.

In contrast the NP approach to palliative care is more holistic;

*Whereas the Nurse Practitioners that I have seen involved have been more focused on you know like what other comfort measures can we provide. What are we doing about you know the spirituality, liaising with the family, you know have we talked to them about what we do when the time comes? [ACF RN]*

### Flow process

NP knowledge of the dual professions (nursing and medical), the systems (residential aged care and acute care) and the resident and family preferences, enabled them to navigate systems and become a connector between professional groups. NPs were able *to speak the same language* as both the doctor and the ACF staff. ACF staff were more confident to talk to the NP because they were nurses, with a common emergent phrases of *it is easier for a nurse to talk to a nurse* and *we speak the same language*. ACF staff, because they couldn’t speak the same language as the doctor, chose instead to contact the NP directly with their concerns requesting the NP act as a mediator between them and the PCP.

*but sometimes they’re [ACF staff] unable to express [their concerns] whereas the Nurse Practitioners can express very clearly to the doctors [ACF manager].*

The specialist diagnostic and referral skills of the NPs meant they were able to refer residents to the ED with recommendations and then follow up with ED thereby reducing ED length of stay.

*…we also work hand in hand with the tertiary, you know, facilities. For example when assessing a patient and we require diagnostics that we cannot order …we send the patient in with the recommendation of what we want…maybe we want an x-ray to rule out a fracture before we continue pain managing patients in their nursing home. So with that in mind the patient can be turned around to us if the x-ray is satisfactory then we will continue managing the patient in their residence [NP]*.

Similarly, from the allied health perspective;

*… if we sent them to hospital … the Nurse Practitioners would make contact and they would oversee the return of the resident [OHP]*.

NPs, because they knew both the capabilities of the ACF, the services each facility could provide and the needs of the resident, were able to assist the family with making an informed decision when accepting placement back into the ACF.

*The hospital social workers were very nice, but … they didn’t have the same knowledge that the NP had, and the hospital social worker doesn’t go to the facilities to see what the facilities have to offer, whereas they [NP] did, so they were able to offer knowledge of that facility, with regards to … basically how much the facilities could offer and whether or not it would suit my mother’s particular condition. And the hospital social workers could not have offered the same amount of knowledge with regards to that [ACF family].*

### Outcomes

The outcomes represent the consequences of the healthcare service model provided and the impacts on resident health, family impacts and quality of life. In this study, structural and process elements both influenced resident/family and health professionals’ perceptions of quality care. The NP service facilitated the ACF resident receiving quality care in a timely manner that meets the resident and/or family wishes. When PCPs were unable to attend, the ‘in reach’ approach (structural dimension) meant the NP’s were able to respond and intervene, prevented unnecessary ED presentations and facilitated timely return to the ACF. The following excerpt is typical of many participant responses.

*I think it [NP service] has contributed really positively in the fact that, um, we can prevent the big upheaval of the person getting so … um, say her UTI that is untreated, she gets so delirious that we have had to send them up to hospital when their system is you know, failing. We can prevent a lot of those admissions and of course I am in the dementia area, so if we can prevent anyone going up to the hospital because they become so confused and disoriented and the relatives are stressed. It is quite an awful event if you have to send someone … up to the hospital. So if we can prevent that, it has a great impact on the residents themselves.*

Keeping the resident at home was important for all concerned. Residents and family alike articulated satisfaction with the NP service for this outcome.

*Mum is very distressed with any changes in her environment at this stage so the last thing I want is her taken out of that environment and put in a hospital environment, which really … and Emergency Department is not a place for a patient with her condition. So if that can be prevented then it's absolutely wonderful [resident family]*.

The process elements of functioning within a nursing model of care and taking time to conduct a thorough assessment, to consult with the resident and family and to develop a collaborative plan of care were major contributors to resident and family satisfaction and HA. HA, in addition to being a desirable outcome for the resident, also saved staff time and healthcare resources. Within this group there was the general belief that transferring the resident took time, and in the greater schema of thing, keeping the resident ‘at home’ saved them time.

*… I think most nurses would much rather manage the residents that they know and be part of that rather than the time it takes to sort of prepare for the hospital and the return from hospital and trying to, you know it is often underestimated how much disruption that causes and how much time that takes out of your day [RN].*

The NP intervention to keep the residents at home also saved time and resources for other healthcare services including the ambulance, ED and inpatient hospital services.

*Then you have the Ambulance taken off the road so you are potentially taking a paramedic off the road to do a transfer and then when they get to the Emergency Department they sit there and chew up the time and resources of Emergency Department. And the number of times that I have seen that happen and then they literally almost have a revolving door and come back within a couple of hours. It is an enormous amount of resources to treat what could have been simply managed by some simple IV antibiotics or you know some sort of simple intervention that a Nurse Practitioner can do [RN].*

Elements of distance, funding and professional restrictions all limited the NPs ability to work to their full potential, having a negative impact on the HA RACS service delivery and patient outcomes.

*Thinking more about what obstacles our role faces I would like to mention availability of cars. We have not really ever been delayed a whole day but the mere fact that we have to share a vehicle means we have to be good at triaging and organising our resources [NP].*

A further comment;

*Distance can be an issue [another ACF in another distant town] have requested regular review for their aged care residents but this has been placed on the back burner as our workload currently has been too busy to fit it in [NP].*

NPs access rebates for a small number of services listed on the Medical Benefits Schedule contributed to a dependence on hospital healthcare services and increased presentations to the ED. The following extract provides an example;

*…sometimes if you have (a resident with) an acute abdomen and you want to determine where to … with your pathway of care…it would be a consideration that you get an abdominal x-ray and so then that makes us reliant on other health care services or other personnel. But it’s not a huge percentage of people but it…I guess it would be the same as residents requiring chest x-rays [NP].*

Quality care, for participants in this study, was about timely intervention, HA, timely return home (to the ACF), partnering with residents and family (knowing what they want) and resident and health professional satisfaction.

## Discussion

The Donabedian framework guided the evaluation of the relationships between the structural, process and outcome dimensions of the aged care NP model of care within an ACF, HA service. Introduction of the NP service in this regional setting is an effective and viable service that has had positive impact on HA, with reported acceptance by residents, their families, ACF staff, PCPs and the NPs themselves.

The structural dimension of how the NP service is implemented, directly and indirectly impacts the provision of an HA service that is timely, responsive and acceptable. NPs assess, detect and intervene early for a range of resident symptoms or treatments that would ordinarily lead to ED transfer, lengthy ED presentation or hospital admission. With estimates of around 30 residential aged care transfers/100 beds/year [33], any reduction will benefit the healthcare system. The process where the NPs in our study responded and intervened early, equiped staff with the skills to manage the event, contribute to seamless coordination of care and the subsequent impact on HA, is mirrored elsewhere in the literature [10, 11, 34, 35].

In this study, the NPs advanced assessment skills and legal authority to prescribe, refer and request diagnostics, influenced the process dimensions, facilitating early assessment and response and minimising hospital transfers. HA was an important outcome for residents, family and health professionals. The NP model of care provides a service that is dynamic, flexible and responsive to both patient and organisational demands within a structure where such service demands were previously absent [36].

Similar to other studies, this study found that the process of referring to and accessing the resident’s primary care physician (PCP) when the resident is deteriorating has its limitations [35, 37–39]. Specifically, failures in the traditional process of PCP referral, negatively impacted on quality outcomes as resident care was delayed while they waited for the PCP to arrive or be transferred to the ED for review. The NP service adequately ‘fills’ this service gap. In this study, the structure of the NPs service supported the NPs ability to function as an independent and autonomous practitioner, hence they were able to triage referrals and respond in a timely manner to meet the needs of all parties. The existing process of referral to the NP service, occurring after attempts to access the PCP were exhausted, is an inhibitor of quality care outcomes. For the PCPs in this and other studies, the NP service provided them with a mechanism to relieve some of their practice pressures whilst still meeting the timely needs of the residents under their care [36].

Increasing knowledge of the existing NP referral structure and reviewing PCP/NP interprofessional collaborations has the potential to increase activation of the NP service and avoid unnecessary resident transfer to the ED. The paucity of healthcare professionals and indeed the general public’s understanding to the NP role [36, 40] impacts on consumer decision to see the NP as well as NPs being able to work at their full scope of practice [41]. ACF staff, PCPs, residents and their families having had experience with the NPs requested referral to the NPs. Thus it is important to re-evaluate the structural dimensions of the NP service and their shared role with the PCP in the management of the aged care resident. Indeed as mutual trust matures, it is important that the resident management model be clearly articulated and allowed to evolve over time [42].

The optimal level of expanded scope of practice and NP role integration into primary health teams is subject to discussion [42]. NPs in this study only had access to four items listed on the Medicare Benefits Schedule, We found that this structural dimension around NP regulation was a barrier to practice, response and referral process. These government regulatory barriers, of limitations in access to Medicare Benefits Schedule, and the impact on the ability of the NPs to work in the system is consistently reported in the literature [6, 27, 36, 43, 44].

NPs bridge professional gaps and establish strong workable, collaborative relationships between the resident, ACF staff, the PCP, the ACF and the acute care sector,0ol contributing to seamless coordinated care for the resident. This interprofessional approach to shared decision making and engagement of family and residents as partners in their own care are key elements of high quality and cost effective health services [45]. Traditions, perceptions of ‘ownership’ of care, making contact with the PCP and the availability of the PCP to respond are all reported in the literature [39]. Interprofessional relationships combined with traditional models of healthcare delivery and limited understanding of the NP role all impacted the referral process and limit the potential benefits of the NP service. Interprofessional differences, particularly those that challenge traditional relationships will and do impact on the referral process and quality outcomes [46]. Hilligoss, argues that when handover (referral in this context) occurs between groups with potentially dissimilar healthcare models, the use of simple mnemonic tools may not be sufficient in helping the different parties to understand the issues at hand from their professional perspective [47]. The NPs in our study were able to bridge this gap. ACF staff drew on the expert communication skills of the NPs to articulate their concerns to the PCPs. Indeed, communicating with and between multidisciplinary groups constituted a significant component of the NPs work [48]. This spill over effect of the HA program and empowering ACF nursing staff to move beyond historical hierarchal structures to actively engage with the PCPs to provide additional nursing services has been reported elsewhere [46].

Integrating best practice into ACFs has it challenges [49]. The NPs delivering a nursing model of care situated within the HA service was instrumental in upskilling ACF staff (nursing and allied health) facilitating their confidence and ability to work to fulfil their scope of practice which in turn positively impacted on workplace satisfaction. Enhanced staff clinical confidence promoted earlier assessment and referral to the NP and ultimately HA, an observation reflected in other studies that report fewer hospital admissions when ACFs employ an NP [50].

ACF staff willingness to engage in best practice conversations will occur when staff establish quality relationships with important peers (or the NP in our study) [51] and actively participate in decision-making. Our findings support the observations of others [49] in that a change in behaviour is more likely when these conversations happen ‘on common ground’ such as within professional groups. ACF staff were responsive to assistance with decision-making and engaging in learning opportunities because the NP ‘*was a nurse’* consequently learning opportunities were predominantly initiated by the staff. These social influences, processes, professional networking and shared decision-making influence the adoption of best practice principles [49]. The process dimension where the NPs actively engage with ACF staff to capacity build, assist with decision-making and support communication pathways with the PCP all contribute to quality outcomes for the resident, staff satisfaction and a positive work environment. Anderson et al. also reported that these local connections that facilitate exchange of new information will contribute to a positive work environment, staff satisfaction and enhanced resident care [51].

Consistent with previous studies, staff want to keep residents at home in the ACF [52, 53] and residents want to ‘stay at home’. HA was important as transferring residents to hospital was viewed as disruptive and confusing for the resident. For the ACF staff, manipulation of the NP referral process was an avenue to meet this objective. Older persons report dissatisfaction with continuity of care following discharge from hospital, and report feelings of disempowerment by the system of care delivery because of failure to be included in the decision-making about their own care [54]. Processes where the NP takes time to listen, explain and engage residents, family and healthcare professionals in decision-making was intrinsic to continuity of care and a coordinated resident journey from the ACF through acute care admission and back to the ACF. This process explains why other health professionals working in close proximity to the NP report feelings of support and collaboration that complement the traditional medical role [8, 13, 55]. Time spent engaging with the NP was closely linked to satisfaction with care and the older person’s perception of quality of care [56, 57].

When a resident requires transfer to an acute care facility, family involvement may be only brief or absent contributing to a fragmented traumatic experience for both the resident and the family [36, 46, 58]. We found the process where the NPs spend time with residents and the family to formulate collaborative plans of care, and actively navigate between professional groups and across healthcare sectors to implement these plans minimised negative aspects transfer experience. This contribution of the NP to the resident’s continuity of care and streamlined approach to care has the potential to reduce unplanned admissions to hospital and facilitate timely return to the facility [36, 59, 60].

Limitations include the fact that this study was conducted in a single region that is serviced by the two NPs in the study. Additionally given the low response rate among some health providers (eg. medical doctors) and aged care residents, bias may have occurred with participants choosing to engage in the research because of a personal predisposition on the topic. These factors, purposive sampling and sample size, whilst consistent with qualitative studies, limits transferability of findings.

## Conclusion

This study provided a methodological approach to evaluate the structural, process and outcomes dimensions of a community based NP service on the quality of care for residents in a regional ACF. Findings from this study indicate that the NP role within the HA service provides a model of care that complements the existing service where the PCP is the primary health provider. We found that the NP, because of their advanced clinical skills and prescribing rights, were able to deliver a range of timely health services within the ACF, saving the PCP time, upskilling and supporting ACF staff to keep the resident at home. Conversely, NPs lack of the access to Medicare Benefits Schedule rebates restricts their scope of practice. Aa a role that is evolving, and as with any service change that challenges traditional professional boundaries and lines of communication, ongoing adjustments and renegotiating referral processes are essential to ensure quality resident outcomes.

## Abbreviations

ACF: Aged care facility
HA: Hospital avoidance
NP: Nurse practitioner
PCP: Primary care physicians
RACS: Residential Acute Care Service
RN: Registered nurse
SPO: Structure, process and outcome.
